# HIV-Induced Type I Interferon and Tryptophan Catabolism Drive T Cell Dysfunction Despite Phenotypic Activation

**DOI:** 10.1371/journal.pone.0002961

**Published:** 2008-08-13

**Authors:** Adriano Boasso, Andrew W. Hardy, Stephanie A. Anderson, Matthew J. Dolan, Gene M. Shearer

**Affiliations:** 1 Experimental Immunology Branch, National Cancer Institute (NCI), National Institutes of Health (NIH), Bethesda, Maryland, United States of America; 2 Department of Immunology, Faculty of Medicine, Imperial College, Chelsea and Westminster Hospital, London, United Kingdom; 3 Henry M. Jackson Foundation, Infectious Disease Clinical Research Program (IDCRP), Wilford Hall Medical Center, Lackland Air Force Base, Texas, United States of America; 4 Henry M. Jackson Foundation, Infectious Disease Clinical research Program (IDCRP), San Antonio Military Medical Center (SAMMC), Fort Sam Houston, Texas, United States of America; New York University School of Medicine, United States of America

## Abstract

Infection by the human immunodeficiency virus (HIV) is characterized by functional impairment and chronic activation of T lymphocytes, the causes of which are largely unexplained. We cultured peripheral blood mononuclear cells (PBMC) from HIV-uninfected donors in the presence or absence of HIV. HIV exposure increased expression of the activation markers CD69 and CD38 on CD4 and CD8 T cells. IFN-α/β, produced by HIV-activated plasmacytoid dendritic cells (pDC), was necessary and sufficient for CD69 and CD38 upregulation, as the HIV-induced effect was inhibited by blockade of IFN-α/β receptor and mimicked by recombinant IFN-α/β. T cells from HIV-exposed PBMC showed reduced proliferation after T cell receptor stimulation, partially prevented by 1-methyl tryptophan, a competitive inhibitor of the immunesuppressive enzyme indoleamine (2,3)-dioxygenase (IDO), expressed by HIV-activated pDC. HIV-induced IDO inhibited CD4 T cell proliferation by cell cycle arrest in G1/S, and prevented CD8 T cell from entering the cell cycle by downmodulating the costimulatory receptor CD28. Finally, the expression of CHOP, a marker of the stress response activated by IDO, was upregulated by HIV in T cells in vitro and is increased in T cells from HIV-infected patients. Our data provide an in vitro model for HIV-induced T cell dysregulation and support the hypothesis that activation of pDC concomitantly contribute to phenotypic T cell activation and inhibition of T cell proliferative capacity during HIV infection.

## Introduction

Infection by the human immunodeficiency virus (HIV) type 1 causes a chronic, progressive and eventually deadly impairment of immune function in humans [1]. Although HIV can infect most CD4-expressing cells, particularly CD4 T helper cells, it is accepted that infection of this cell subset cannot solely explain the HIV-associated immunopathogenesis [2], [3]. Multiple evidence supports the hypothesis that the key events of HIV pathogenesis reside in the complex interactions between the virus and the immune system, for example: 1) the frequency of infected CD4 T cells is too low to uniquely account for their depletion and dysfunction [4], [5]; 2) a large proportion of circulating virus is estimated to be non productively infectious, but may still interact with the immune system [6], [7]; 3) cells that are not susceptible to infection, such as CD8 T cells and B cells, also show signs of functional impairment [8], [9]; and 4) signs of chronic immune activation, such as expression of T cell activation markers and lymphoadenopathy, are observed during the infection and correlate with disease progression [10]–[13].

The impairment of T cell responses in HIV infected patients has been described both in vitro and in vivo [14], [15]. HIV-infected patients show reduced delayed hypersensitivity skin test reactions and inefficient responses to common vaccines [15], [16]. The in vitro proliferative response to recall antigens is lost early during infection, and is followed by loss of response to alloantigens and mitogens [14]. The loss of T cell function progresses during the course of HIV disease, and is predictive of AIDS onset and death [14], [15]. Immune exhaustion, caused by chronic T cell activation, is one of the most supported theories which could explain the HIV-associated immunodeficiency [13], [17]. Compelling evidence for the role of chronic T cell activation in HIV pathogenesis comes from the observation that not only the expression of certain surface activation markers, such as CD69 or CD38, is increased on T cells from HIV-infected patients [18], [19], but also that the frequency of CD38^+^ CD8 T cells is the best predictor of disease progression, better than plasma viral load and CD4 count [10], [12]. Furthermore, signs of T cell activation are not observed in natural hosts of HIV and simian immunodeficiency virus (SIV), species that do not develop immunologic disease despite high levels of viral replication [13], [20].

Several mechanisms are thought to contribute to T lymphocyte activation and exhaustion. Of these, the two most frequently cited are 1) the constant antigenic stimulation caused by HIV replication, combined with the lymphotropic nature of the virus, which may chronically trigger T lymphocytes [13], [17]; and 2) the presence of circulating immunostimulatory factors of microbial origin, such as lipopolisaccaride, which translocate from the damaged gut mucosa during the early phases of acute infection and may provide a constant stimulus for T cells and innate immunity [17], [21], [22]. The extent of the contribution of both these mechanisms, and of others, is unclear and is subject of discussion. The reactivation of latent viral infections, such as CMV or EBV, due to the reduction of immune control, as well as the occurrence of other opportunistic infections, may also provide further antigenic stimulation to T cells [23]–[26]. On the other hand, chronic activation of innate immunity may derive not only from microbial bioproducts [17], [22], but also from direct activation of plasmacytoid dendritic cells (pDC) by HIV [27].

pDC are important players in innate immune responses against viral infections [28], [29]. They can be activated by HIV in a CD4-dependent manner to produce large amounts of type I interferon (IFN) [30], [31], and to express the tryptophan-catabolizing enzyme indoleamine-2,3-doxygenase (IDO) [32]. As an alternative to the T cell activation hypothesis, we recently proposed that the chronic activation of pDC by direct interaction with infectious and noninfectious HIV particles may be the main driving force for HIV disease progression [27]. Thus, the proapoptotic effect of type I interferon, combined with the immunesuppressive function of IDO and its interaction with regulatory T cells, may contribute to both the progressive depletion of CD4 T cells and their functional impairment [27], [33]–[35]. It is noteworthy that high IFN-α production and IDO activity are detected both in the circulation and in lymphoid tissues during HIV/SIV infection [31], [32], [36]–[39].

Here we show that in vitro HIV-induced IFN-α production stimulates an activated phenotype in both CD4 and CD8 T cells, characterized by increased expression of CD69 and CD38. However, T lymphocytes from HIV-exposed peripheral blood mononuclear cells (PBMC) are unresponsive to subsequent T cell receptor (TCR) stimulation, and this unresponsiveness is partially mediated by HIV-induced IDO activity. We then describe, using a two-step experimental protocol, that HIV-stimulated IDO affects T cell activity by arresting CD4 T cells in the G1 phase of the cell cycle, and by inducing CD28 downregulation by CD8 T cells. Finally, we show that the expression of CHOP, a marker of the stress-response system activated by IDO-induced tryptophan deprivation [40], is increased in vitro by HIV and is elevated in lymphocytes from HIV-infected patients.

## Results

### HIV-induced type I IFN upregulates the T cell activation markers CD69 and CD38

**Figure 1:**
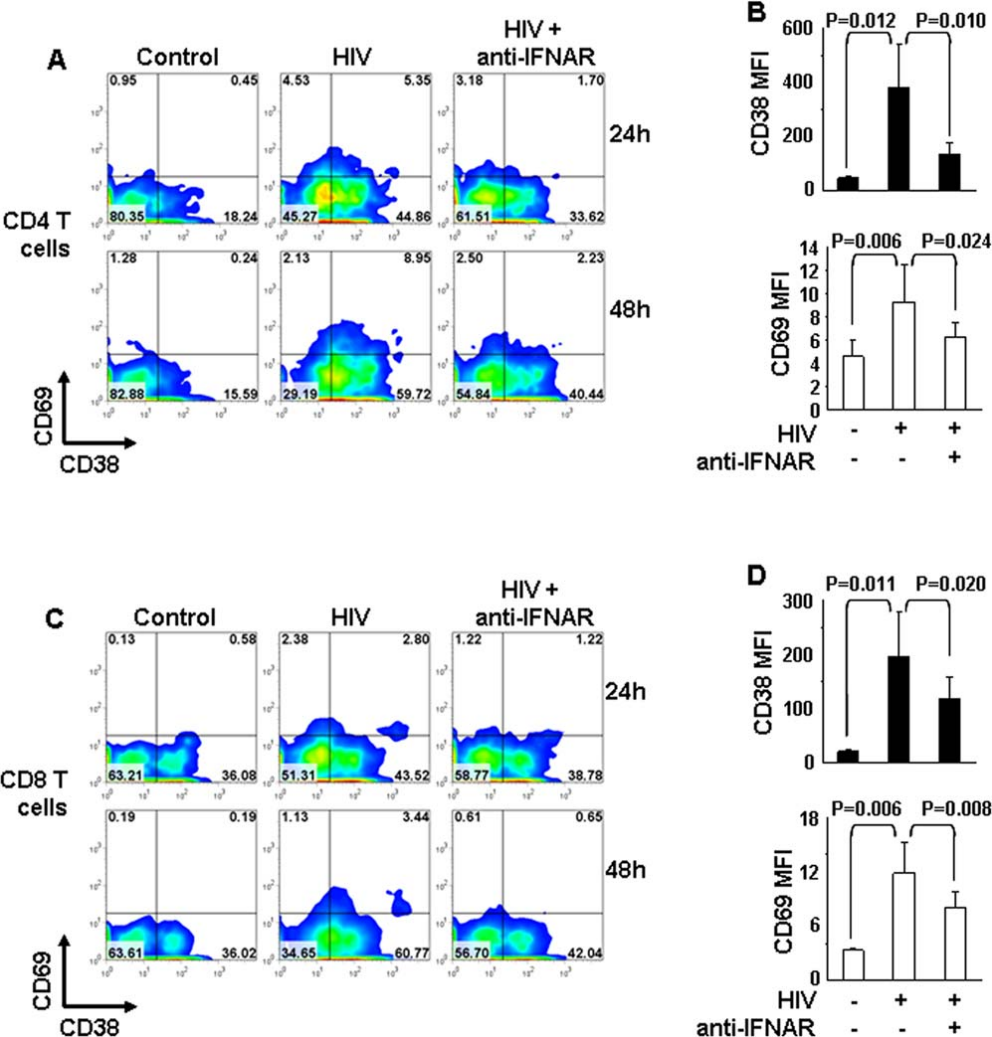
HIV induces increased CD69 and CD38 on T cells in a type I IFN-dependent manner. PBMC from HIV-uninfected donors were cultured for 24 and 48 hours in presence of control microvescicles, HIV alone or in presence of blocking antibodies against the cellular receptor for IFN-α (anti-IFNAR). CD38 and CD69 expression were analyzed by flow cytometry on gated CD3^+^CD4^+^ and CD3^+^CD8^+^ cells (CD4 and CD8 T cells, respectively). (A) and (C) show flow cytometry contour plots of CD69 and CD38 expression for one example experiment for CD4 and CD8 T cells, respectively. (B) and (D) show bar graphs summarizing mean fluorescence intensity (MFI) of CD38 and CD69 in CD4 and CD8 T cells, respectively (48 hours only). Mean values±standard error calculated on 5 independent experiments are shown in the bar graphs.

Elevated expression of certain surface markers is regarded as a hallmark of chronic T cell activation during HIV infection and is predictive of disease progression [10], [12], [18], [19]. We tested whether direct exposure of PBMC from HIV-uninfected donors to infectious or RT-deficient (AT-2) HIV would affect expression of the activation markers CD69 and CD38 on CD4 and CD8 T cells. Flow cytometry analysis revealed a significant increase in CD69 and CD38 on CD4 (Fig. 1A and 1B) and CD8 T cells (Fig. 1C and 1D) after 24 and 48 hours of incubation with HIV, measured both as proportion of marker-expressing cells (Fig. 1A and 1C) and mean fluorescence intensity (MFI) (Fig. 1B and D). Because antigen recognition and T cell receptor (TCR) engagement are unlikely to occur in this in vitro setting within 24 hours, we reasoned that the mechanism of CD69 and CD38 induction would be independent of classic T cell activation.

**Figure 2:**
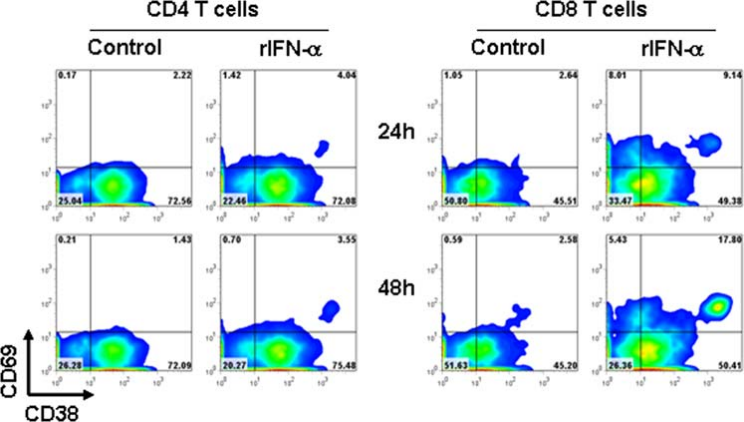
rIFN-α induces increased CD69 and CD38 on T cells. PBMC from HIV-uninfected donors were cultured for 24 (upper panels) and 48 hours (lower panels) in presence or absence of recombinant IFN-α (rIFN-α). CD38 and CD69 expression were analyzed by flow cytometry on gated CD3^+^CD4^+^ and CD3^+^CD8^+^ cells (CD4 and CD8 T cells, respectively). Flow cytometry contour plots of CD69 and CD38 expression for one example experiment for CD4 (left panels) and CD8 T cells (right panels).

Type I IFN is rapidly produced by pDC upon exposure to HIV and can directly affect T cell phenotype and function [30], [31], [41]. In the present study IFN-α levels in supernatants of HIV-exposed PBMC were 438.5±208.9 pg/ml and 511.2+237.1 pg/ml after 24 hours and 48 hours, respectively. IFN-α was below 40 pg/ml in supernatants of untreated PBMC at both time points. We tested whether type I IFN may contribute to the HIV-induced expression of CD69 and CD38. Blocking antibodies directed against the subunit 2 of the IFN receptor (anti-IFNAR), specific for IFN-α, largely prevented the induction of CD69 and CD38 on CD4 (CD69 and CD38 MFI reduction of 75±8% and 62±19%, respectively) (Fig. 1A and 1B) and CD8 T cells (CD69 and CD38 MFI reduction of 44±8% and 45±2%, respectively) (Fig. 1C and 1D) after 24 and 48 hours. No effect was observed when antibodies against the receptor subunit specific for IFN-γ (anti-IFNGR) were used (data not shown). Furthermore, culture of PBMC from HIV-uninfected donors in the presence of recombinant human IFN-α resulted in increased CD69 and CD38 expression on CD4 (Fig. 2, left panels) and CD8 T cells (Fig. 2, right panels), similar to that observed after exposure to HIV.

These data collectively suggest that HIV-induced type I IFN may contribute to the maintenance of a chronic activated T cell phenotype, even in the absence of classic antigenic triggers for T cell activation.

### In vitro exposure to HIV impairs proliferative T cell responsiveness, role of HIV-induced IDO

**Figure 3:**
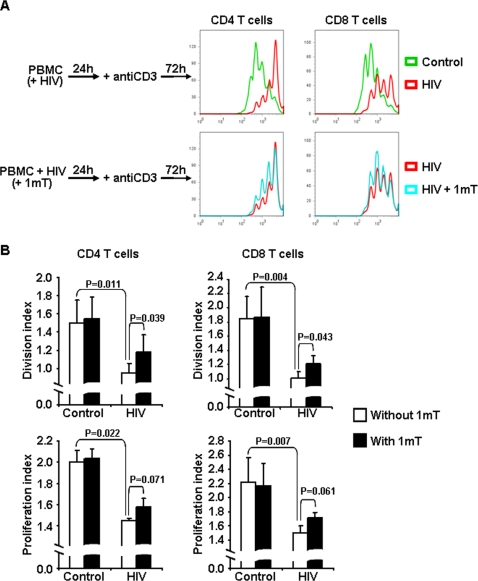
HIV exposure impairs T cell proliferative responses, contribution of IDO-mediated tryptophan catabolism. CFSE-labeled PBMC from HIV-uninfected donor were cultured for 24 hours in presence of control microvescicles, HIV alone, or in presence of the IDO inhibitor 1-methyl-D-tryptophan (1mT). After 24 hours anti-CD3 was added to the cultures and cells were analyzed by flow cytometry after 72 hours. (A) Flow cytometry histograms showing CFSE dilution for one example experiment for CD4 (left panels) and CD8 T cells (right panels) are shown. Upper panels show the comparison between control-pretreated cells (green line) and HIV-pretreared cells (red line); bottom panels show the comparison between HIV-pretreared cells (red line) and cells pretreated with HIV in presence of 1mT (Blue line). One representative of 5 independent experiments is shown. (B) Bar graphs showing division index (number of cell divisions/total cell number) and proliferation index (number of cell divisions/number of divided cells) of CD4 (left panels) and CD8 T cells (right panels) pretreated with AT-2 HIV or mock and stimulated with anti-CD3 in presence (solid bars) or absence (open bars) of 1mT. Mean values±standard error calculated on 5 independent experiments are shown.

We tested whether the induction of an activated phenotype on CD4 and CD8 T cells after exposure to HIV corresponded to increased proliferative responses. CFSE-labeled PBMC from three HIV-uninfected donors were cultured in the presence or absence of HIV for 24 hours. These cells were then stimulated with anti-CD3 (OKT3) antibodies. Proliferation of CD4 and CD8 T cells was evaluated after 3 days by CFSE dilution. Pre-treatment with HIV significantly inhibited proliferation of both CD4 and CD8 T cells, expressed by both division and proliferation indices (Fig. 3A upper panels and Fig. 3B). Because we previously reported that HIV induces pDC to express the immunosuppressive enzyme IDO [32], we tested whether blockade of IDO with the competitive inhibitor 1mT would counteract the anti-proliferative effect of HIV exposure. Preincubation of PBMC with 1mT partially prevented the proliferative defect induced by HIV in CD4 and CD8 T cells (Fig. 3A lower panels and Fig. 3B). Statistically significant 1mT-induced increases were observed for both CD4 and CD8 T cells in the division indices, while increases in the proliferation indices only approached statistical significance (Fig. 3B). Multiple downregulatory mechanisms may be activated by HIV and act alongside IDO in suppressing T cell responses. For example, HIV-mediated induction of CD4 T cell apoptosis [41], of the negative regulator PDL-1 [42]–[44] and of regulatory T cell survival [39] may all affect T cell responses in an IDO-independent way.

**Figure 4:**
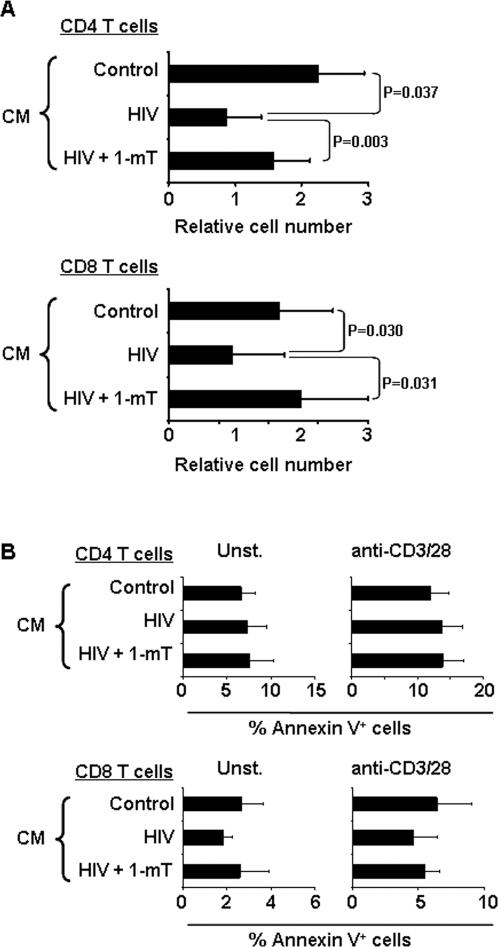
HIV-induced IDO suppresses both CD4 and CD8 T cell proliferation in a 2-step experiment. (A) CD4 and CD8 T cell proliferation is shown as the increase in the number of viable cells measured using a bioreduction assay. Relative cell number was calculated for each sample as ratio between stimulated (with anti-CD3 and anti-CD28) and unstimulated cells cultured in the three different conditioned media (CM). (B) CD4 and CD8 T cell apoptosis is shown as frequency of annexin V+ cells measured by flow cytometry. Apoptosis of cells cultured in the three different conditioned media (CM) is shown for both unstimulated and stimulated (with anti-CD3 and anti-CD28) cells. In all cases mean values±standard error calculated on 8 independent experiments are shown.

To better investigate the contribution of IDO to HIV-induced impairment of T cell responses, we designed a 2-step experimental model. Using this experimental design, T cells were exposed to an HIV-induced tryptophan-depleted environment, while their direct contact with pDC and monocytes which express proapoptotic and antiproliferative molecules such as PDL-1 and tumor necrosis factor family members was limited. We cultured PBMC from HIV-uninfected donors in the presence or absence of HIV, with or without 1-mT, for 48 hours. Supernatants were then collected and used as conditioned media (CM: control, HIV, HIV+1mT), as described in Material and Methods. We used these CM to culture autologous CD4 and CD8 T cells, in the presence or absence of anti-CD3 (OKT-3) and anti-CD28 antibodies (complete absence of pDC from CD4 T cells is shown in Supplemental Figure S1). After three days, proliferation was evaluated as increase in the number of viable cells, measured using a bioreduction colorimetric assay. Anti-CD3/28-induced proliferation was reduced in CD4 and CD8 T cells cultured in HIV CM compared to both control CM and HIV+1mT CM (Fig. 4A). Control experiments were performed by culturing CD4 T or CD8 T cells in fresh media, in the presence or absence of HIV or HIV plus 1mT, to distinguish between the effect of tryptophan depletion and the direct cytopathic effect of HIV which may still be present in the CM. Direct exposure to HIV showed no significant effect on the proliferative response of CD4 T and CD8 T cells, nor did addition of 1-mT (data not shown). Our in vitro model demonstrates that HIV-induced tryptophan catabolism has a direct negative effect on CD4 and CD8 T cell proliferative responses.

### The antiproliferative effect of IDO is not associated with increased apoptosis

We analyzed the induction of apoptosis in CD4 and CD8 T cells cultured in the CM. The percentage of cells expressing the apoptotic marker annexin V was measured by flow cytometry. Culture in HIV CM or HIV+1mT CM did not result in increased apoptosis of CD4 or CD8 T cells compared to control CM (Fig. 4B), suggesting that HIV-induced, IDO-mediated inhibition of T cell proliferation is not due to increased apoptosis.

### HIV-induced tryptophan catabolism inhibits CD4 and CD8 T cell proliferation at different stages of the cell cycle

**Figure 5:**
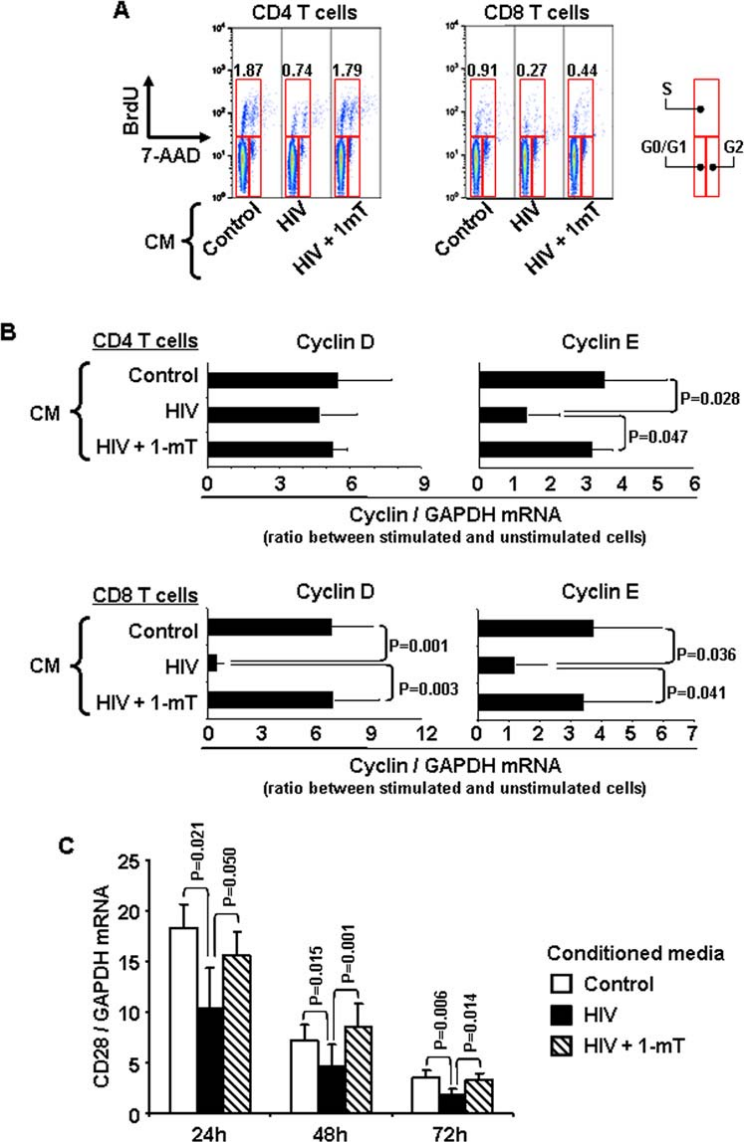
Effect of HIV-induced IDO on CD4 and CD8 T cell cycle progression in a 2-step experiment. (A) Flow cytometry dot plots showing BrdU incorporation and DNA staining with 7-AAD for one example experiment for CD4 and CD8 T cells stimulated with anti-CD3 and anti-CD28 in the three different conditioned media (CM); red boxes indicate gates for cells in G0/G1, S and G2 phase; numbers represent the percentage of T cells in S phase for each condition. (B) Cyclin D1 (marker of G1 phase) and cyclin E1 (marker of S phase) mRNA expression in CD4 and CD8 T cells stimulated in the three different conditioned media (CM). Relative mRNA levels are calculated as ratio between stimulated (with anti-CD3 and anti-CD28) and unstimulated cells. (C) CD28 mRNA expression in unstimulated CD8 T cells cultured in the three different conditioned media (CM) over a 72 hours period. In all bar graphs mean values±standard error calculated on 8 independent experiments are shown.

We tested whether cell cycle progression was affected in CD4 and CD8 T cells stimulated in the CM. BrdU incorporation and DNA content (7-AAD staining) were analyzed by flow cytometry. The percentage of both CD4 and CD8 T cells in the G0/G1 phases of the cell cycle was increased, whereas that of cells in the S phase was decreased when cells were stimulated in HIV CM compared to both control CM and HIV+1mT CM (Fig. 5A). Because the BrdU/7-AAD staining does not permit precise discrimination between cells stationing in G0 and cells that have entered the cell cycle but are stopped in the G1 phase, we analyzed mRNA expression for cyclin D1 (typically expressed in G1 phase) and cyclin E1 (typically expressed in late G1 and S phase). We found that CD4 T cells upregulated cyclin D1 expression in HIV CM at levels comparable to control CM and HIV+1mT CM. However, CD4 T cells cultured in HIV CM failed to upregulate cyclin E1 (Fig. 5B, upper panels). Conversely, upregulation of both cyclin D1 and cyclin E1 was impeded in CD8 T cells by culture in HIV CM, compared to both control CM and HIV+1mT CM (Fig. 5B, lower panels).

Downregulation of CD28 expression has been reported for CD8 T cells from HIV-infected patients [45], [46], and has been described as a potential consequence of tryptophan starvation in a murine model [47]. Therefore, we tested whether CD28 mRNA expression in CD8 T cells was affected by culture in the CM. We found a progressive decrease of CD28 mRNA expression by CD8 T cells cultured for 3 days in any of the three CM (Fig. 5C). Such decrease was significantly enhanced in HIV CM, compared to both control CM and HIV+1mT CM (Fig. 5C). No alteration of CD28 expression was observed in CD4 T cells cultured in HIV CM compared to control CM (data not shown)

These data collectively suggest that HIV-induced tryptophan catabolism has different effects on CD4 and CD8 T cells, resulting in the arrest of CD4 T cells at the G1/S transition checkpoint of the cell cycle and in the downregulation of CD28 expression by CD8 T cells, which eventually prevents their entry into the cell cycle due to insufficient costimulation.

### HIV activates the GCN2-stress-response system in vitro and in vivo

**Figure 6:**
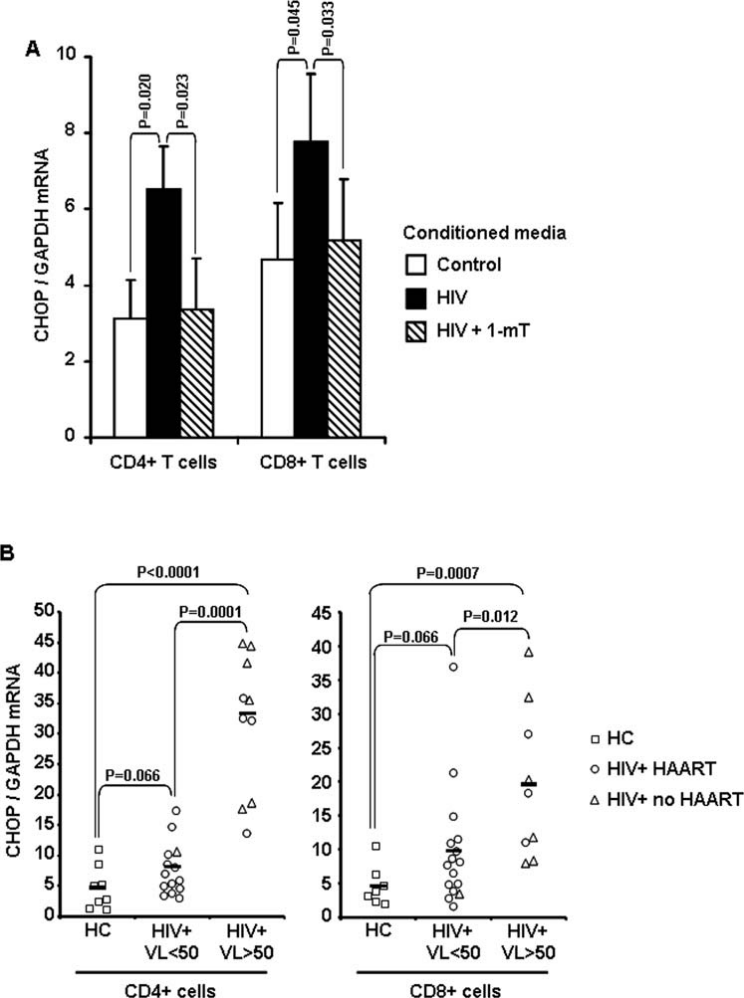
CHOP mRNA expression is upregulated in CD4 and CD8 T cells by HIV-induced IDO in vitro and in HIV-infected patients in vivo. (A) CHOP mRNA expression in unstimulated CD4 and CD8 T cells cultured in the three different conditioned media (CM). Mean values±standard error calculated on 8 independent experiments are shown. (B) Plots showing CHOP mRNA expression in CD4^+^ and CD8^+^ cells isolated from PBMC of uninfected healthy controls (HC) and HIV-infected patients (HIV+) with plasma virus levels below (VL<50) or above (VL>50) the detection threshold. Each symbol represents one individual patient or donor: HC are indicated with squares; HIV-infected patients undergoing HAART are indicated with circles (independently of plasma virus load) and HIV-infected patients not undergoing HAART are indicated with triangles (independently of plasma virus load). Horizontal bars represent mean values for HC, HIV+ VL<50 and HIV+ VL>50, respectively.

The immediate effect of tryptophan depletion at the molecular level is the increase in cytoplasmic uncharged tRNA^trp^ [40]. Similar to other cell types, T lymphocytes are sensitive to changes in uncharged tRNA levels, which serve as a monitor for availability of free amino acids [40]. Accumulation of uncharged tRNA^trp^ results in activation of the stress-response system regulated by the GCN2 kinase [40]. The CHOP gene (also known as gadd153) is a well-accepted marker for GCN2 activation [40]. We analyzed CHOP mRNA expression in CD4 and CD8 T cells cultured in the CM. CHOP expression was increased in both CD4 (2-fold) and CD8 T cells (1.5-fold) cultured in HIV CM compared to both control CM and HIV+1mT CM (Fig. 6A). We then tested CHOP mRNA expression in CD4^+^ and CD8^+^ cells isolated from fresh PBMC of HIV-infected individuals (HIV+) and uninfected blood bank donors (HC). Both CD4^+^ and CD8^+^ cells from HIV+ patients with detectable plasma virus level (>50 copies/ml) expressed significantly higher levels of CHOP compared to HIV+ patients with undetectable plasma virus level (<50 copies/ml) and HC (Fig. 6B). Only a trend to increased CHOP mRNA, approaching statistical significance, was observed in HIV+ patients with undetectable plasma virus level (<50 copies/ml) compared to HC (Fig. 6B). Increased CHOP expression did not appear to be directly connected with lack of HAART regimen, but rather with viral replication, measured as plasma virus load (compare triangles and circles in Fig 6B). These results demonstrate that HIV activates the GCN2 stress response system in CD4 and CD8 T cells in an IDO-dependent manner, and that signs of GCN2 activation are observed in lymphocytes from HIV-infected patients in whom viral replication is highly active.

## Discussion

The underlying causes of HIV-driven immunedeficiency are still poorly understood. The high level of expression of T cell activation markers detected in HIV-infected patients during disease progression, together with the fact that expression of such markers is not altered in natural resistant hosts of HIV/SIV, has attracted attention by the scientific community [3], [10], [12], [13], [20], [48], [49]. Although viral replication is considered to be responsible for the chronic T cell activation, the cellular and molecular bases of HIV-induced T cell exhaustion are not fully understood. Based on the growing body of evidence showing potential pathogenic effects of HIV-induced activation of pDC, we recently proposed that chronic activation of innate immune responses, rather than direct T cell activation, may play a major role in HIV pathogenesis [27]. Here we provide a link between HIV-induced activation of innate and adaptive immunity, by showing that signs of T cell activation can be induced by HIV-triggered production of IFN-α. Furthermore, we show that impairment of T cell proliferative responses induced by HIV can be replicated in vitro by exposure of uninfected leukocytes to the virus, and that at least part of this dysfunction is dependent on IDO-mediated tryptophan catabolism.

Our findings presented here raise the possibility that phenotypic signs of CD4 and CD8 T cell activation can be induced by HIV in the absence of antigen presentation or TCR engagement. Both the activation marker CD69 and CD38, commonly used to define the chronically activated status of T cells from HIV-infected patients [10], [12], [18], [19], were increased in a type I IFN-dependent manner by exposure to HIV. Although we cannot exclude that other immunologic pathways triggered by HIV may contribute to the induction of a T cell activated phenotype in vivo, our results that rIFN-α is sufficient to upregulate CD69 and CD38, combined with the reports of elevated levels of type I IFN in plasma and tonsils of HIV-infected patients [22], [38], [41], suggest that IFN-α contributes to inducing and maintaining the activated T cell phenotype characteristic of HIV disease progression. In vitro induction of CD38 and CD69 was described in response to different TLR ligands, including TLR9 agonists [50], suggesting that stimulation of innate immune responses through different routes may contribute to the activated T cell phenotype observed in HIV-infected patients. The hypothesis recently proposed by Brenchley and colleagues on microbial translocation fits well in this view, in that lipopolisaccaride (LPS) and other microbial components, systemically mobilized from the gut, may function as chronic stimuli resulting in phenotypic T cell activation [22]. However, our findings presented here suggest that even in the absence of other microbial components, either derived from opportunistic infections or translocated from the damaged gut, HIV-mediated activation of innate immune responses, particularly those associated with type I IFN production, may be sufficient to trigger the appearance of an activated T cell phenotype. Although a direct correlation between plasma type I IFN and expression of phenotypic activation markers has not been reported in HIV-infected patients, both these parameters have been described to correlate with plasma virus levels or CD4 count [18], [19], [34], [51], [52], suggesting a tight connection among T cell activation, type I IFN production and disease progression.

Similar to what observed in lymphocytes from HIV-infected patients, CD4 and CD8 T cells from in vitro HIV-exposed PBMC showed impaired proliferative capacity when stimulated through classic TCR signalling (anti-CD3 and anti-CD28). Thus, despite carrying phenotypic signs of activation, T cells from HIV-exposed leukocytes have impaired proliferative capacity, which is a key feature of HIV disease [14]–[16]. A similar combination of immunedeficiency despite phenotypic signs of T cell activation was described in mice treated with repeated administration of CpG oligonucleotides, which are powerful activators of pDC [53]. Of note, CpG-treated mice also showed severe lymphoadenopathy and T cell depletion, two other alterations normally observed during HIV disease [53]. All of these symptoms appeared to be milder or absent in IFNAR knock-out mice, demonstrating that, whereas brief activation of pDC may potently enhance the induction of efficient T cell responses [54], prolonged hyperactivation of type I IFN signalling may have deleterious effect on the adaptive immune system [27], [53].

It is still controversial whether type I IFN production is increased or reduced during HIV infection. Reports documenting decreased type I IFN responses after in vitro stimulation of leukocytes from HIV-infected patients compared to uninfected controls suggest that pDC function is impaired [55]–[58]. However, these reports are partially influenced by the reduced frequency of pDC in the circulation during HIV infection which could be a consequence of the relocation of these cells to lymphoid organs [38], [59]–[61]. Although two distinct studies failed to identify pDC in lymphoid tissues of HIV infected patients with low CD4 counts and in macaques infected by SIV in end stage, none of them analyzed the actual levels of type I IFN in the tissues, making it hard to interpret their conclusions [62], [63]. In addition, a strong increase of pDC density in the T cell zone in lymph nodes of asymptomatic HIV infected patients was reported in an earlier study [64], supporting the hypothesis that pDC may relocate to lymphoid tissues at least in asymptomatic stage. Furthermore, it has been recently shown that pDC are indeed chronically stimulated in HIV-infected patients and produce type I IFN, which in turn contributes to their hyporesponsiveness to subsequent in vitro stimulation, probably through a negative regulatory feedback mechanism [65]. The elevated expression of type I IFN-inducible genes at both peripheral and tissue level in HIV-infected patients is also evidence of chronic production of these cytokines [38], [66]. The question can be raised as to why HIV is, compared to other viruses, particularly efficient in inducing chronic pDC activation. We proposed that the expression of CD4, the main receptor for HIV, on the surface of pDC, may render them particularly susceptible to HIV-induced activation [27]. Thus, gp120-CD4 interaction is required for endocytosis of HIV by pDC and subsequent triggering of TLR7 [30], [31], [67] and the association of CD4 to a clathrin-dependent endocytotic machinery [68], [69] may greatly facilitate the endocytosis of HIV and subsequent pDC activation [27]. Moreover, monocytes and macrophages have been shown to produce type I IFN and express IDO when exposed to or infected by HIV, or when exposed to HIV-derived proteins [70]–[74]. Thus, alternative sources of both type I IFN and IDO may contribute to the described mechanisms during HIV infection.

We previously demonstrated that HIV-induced type I IFN contributes to CD4 T cell apoptosis by inducing expression of pro-apoptotic molecules of the tumor necrosis factor (TNF) superfamily [31], [41]. The HIV CM used in this study contain type I IFN in concentrations comparable to those required for the induction of CD4 T cell apoptosis [31], [41], and HIV viral particles may still be present in the same HIV CM. However, in the present study, we did not observe any increase in CD4 T cell apoptosis in the two-step experiment when HIV CM was used. This apparent contradiction is explained by the fact that type I IFN is sufficient and necessary to induce expression of apoptotic ligands, such as TNF-related apoptosis-inducing ligand (TRAIL), but not of their cellular death receptors (DR), such as DR5, which are required for CD4 T cell apoptosis [31], [34], [41]. Of note, in the present study, direct exposure of unseparated PBMC to HIV for 24 or 48 hours resulted in CD4 T cell apoptosis, similar to what described in our previous reports [31], [34], [41]. Engagement of CD4 expressed on T cells by gp120 and/or the contribution of other cellular subsets may be required to result in DR5 expression by CD4 T cells and subsequent apoptosis [34]. One example of these accessory cells is that of pDC themselves, which are completely absent from the CD4 T cell culture in this study (see Supplemental Figure S1) and can express TRAIL and induce CD4 T cell apoptosis when exposed to HIV or type I IFN [67]. The two-step experiment described in this study was designed for the purpose of focusing our analysis on HIV-induced, IDO-mediated tryptophan catabolism, thus limiting the interference by other mechanisms triggered by HIV, including those involving CD4 T cell apoptosis.

The pleiotropic effects of type I IFN and other immunologic mechanisms induced by HIV exposure may all contribute to the suppression of proliferative responses. Here we focused on the catabolism of tryptophan through the enzymatic reaction catalized by IDO, which we previously described to be induced by HIV in pDC through a mechanism that is independent of production of either type I or type II IFN [32], which are the major known inducers of IDO in both human and murine cells [75]. We found that HIV-induced IDO is partially responsible for the proliferative impairment of T cells, and that it differentially affects CD4 and CD8 T cells. The fact that inhibition of IDO had only a limited beneficial effect on CD4 and CD8 T cell proliferation is in accordance with our previously published data, showing a significant albeit limited recovery of proliferative responses by CD4 T cell from HIV^+^ patients [32]. It should be noted that the extent of the proliferative defect and of its correction by IDO blockade may depend in vivo on the levels of viral replication and on the number of circulating CD4 T cells. Indeed, we previously reported that the increase in proliferation induced by 1-mT in PBMC from HIV^+^ patients directly correlates with the CD4 count [32]. Importantly, several reports have described elevated IDO expression in lymphoid tissues, including tonsils, lymph nodes, spleen and the gut-associated lymphoid tissue, during different stages of HIV and SIV infection [36], [37], [39], [76], [77], suggesting that the effects of tryptophan depletion on T cells may be enhanced in lymphoid tissues.

A recent study has identified an isoform of IDO, named IDO2, which appears to be the preferential target of the D-isomer of 1mT we used in the present study [78]. Notably, IDO2 has reduced enzymatic activity compared to IDO, but exerts potent immunesuppressive effect, similar to IDO, which can be inhibited by D-isomeric 1mT [78]. It is therefore likely that part of the recovery of T cell proliferation observed in the present study in presence of 1mT is the consequence of blockade of IDO2, rather than IDO. However, we have previously used D-isomeric 1mT to successfully inhibit in vitro HIV-induced tryptophan degradation and kynurenine production [32], suggesting that this molecule has a significant effect on tryptophan catabolism, whether mediated by IDO or IDO2. Furthermore, in the same study, D-isomeric 1mT was efficient in improving in vitro proliferative responses of T cells from HIV-infected patients [32], confirming its biologic activity. It is unclear whether the immunoregulatory activity of IDO (or IDO2) is the consequence of tryptophan depletion, accumulation of kynurenine bioproducts or a combination of both mechanisms. The limitation of tryptophan availability appears to be sufficient to induce activation of the GCN2 stress response system, which is normally triggered by amino acid depletion[40]. However, Fallarino and colleagues reported that both reduction of tryptophan and addition of downstream bioproducts of the kynurenine pathway are required to alter CD4 and CD8 T cell phenotype and function in a GCN2-dependent manner [47]. The use of 1mT to interfere with HIV-induced IDO activity has the great advantage of limiting both mechanisms, inhibiting tryptophan conversion into kynurenine [32]. On the contrary, supplementation of exogenous tryptophan does not have any effect on kynurenine production. Furthermore, two clinical trials are currently enrolling volunteers for studying the safety of administration of the D-isomeric 1mT for the treatment of metastatic or refractory solid tumors (clinicaltrials.gov; identifier NCT00567931 and NCT00617422), rendering this molecule a good candidate for testing its potential efficacy for improving the immune function in HIV-infected patients in the future.

We and others have previously reported that CD4 and CD8 T cells are differently affected by tryptophan catabolism [32], [47], [79]. In the present study we observed that both cell types are negatively affected by HIV-induced IDO. CD4 T cells are activated by TCR signalling to enter the G1 phase of the cell cycle but cannot progress further through the S phase. In contrast, CD8 T cells downregulate CD28 expression which deprives them of the costimulatory signal during TCR engagement, therefore preventing their entry into the cell cycle. CD28 downregulation is characteristic of CD8 T cells from HIV-infected patients, and contributes to their limited responsiveness to viral antigens, including against HIV [45], [46]. Our data presented here, together with similar findings obtained in a murine model [47] suggest that HIV-induced tryptophan catabolism may be at least partially responsible for CD28 downregulation on CD8 T cells from HIV-infected patients. The in vitro effect of HIV-induced IDO on both CD4 and CD8 T cells was associated with increased expression of CHOP, symptomatic of activation of the GCN2-mediated stress response. Remarkably, such increase was still detectable in circulating CD4^+^ and CD8^+^ cells from HIV-infected patients in whom viral replication was active.

Our previous report indicated that the in vitro proliferative defect of CD4, but not CD8 T cells from HIV-infected donors could be corrected by addition of 1mT [32], which is in apparent contrast with the IDO effect on both CD4 and CD8 T cells that we described here. However, in the in vitro system employed in the present study, 1mT is used to prevent HIV-induced tryptophan depletion rather than correct the existing impairment. It is therefore possible that simple addition of 1mT to PBMC cultured ex vivo from HIV-infected patients [32] may not restore CD28 expression and proliferative response on CD8 T cells, but may be sufficient to release the block on CD4 T cell cycle progression. In addition, other immunologic mechanisms have been described that suppress both CD4 and CD8 T cell responses in HIV-infected patients, whereas the present study was designed with the precise purpose of isolating the effects of tryptophan catabolism from other HIV-induced dysfunctions.

The effect of IDO-mediated tryptophan catabolism on CD4 T cell cycle progression provides a potential advantage for HIV infection and persistence. HIV efficiently infects cycling CD4 T cells, but is incapable of completing reverse transcription in quiescent cells stationed in the G0 phase of the cell cycle [80]. Interestingly, arrest of the cell cycle in the late G1 phase does not interfere with reverse transcription [80], but progression through the cell cycle is required for the production of new viruses [81]. Thus, CD4 T cells which are arrested in the G1 phase by HIV-induced IDO may represent a target for HIV infection, but not a source of new viruses. Such cells could be frozen in a stage in which HIV proviral DNA is safely integrated in the genome, but the lack of production of viral proteins may prevent their recognition by HIV-specific cytotoxic T lymphocytes. We raise the possibility that CD4 T cells arrested in the G1 phase of the cell cycle may contribute to the “hidden reservoir” of HIV-infected cells which persists through the course of infection. Other cell cycle alterations have been described for T cells from HIV infected patients, which may be caused by mechanisms other than IDO and contribute to the same effect of maintaining a pool of infected, inactive cells [82]–[84].

Our results provide the first evidence that phenotypic activation markers can be induced by HIV on human T cells without the need for productive infection or antigenic stimulation, but simply by inducing type I IFN production. Such phenotypically activated T cells have reduced expansion ability, and part of this impairment is due to IDO. pDC have been largely described to be the cellular source of both IDO and IFN-α/β [30], [32], [41], [85], suggesting that chronic stimulation of these mediators of innate immune responses may contribute to both proliferative impairment and phenotypic activation of T cells during HIV infection. The experimental design on which this study is based may represent a simple in vitro model for HIV immunopathogenesis, which may be suitable for testing candidate blockers of the interaction between HIV and immune cells for their immunotherapeutic potential.

## Materials and Methods

### Isolation and culture of blood leukocytes

Blood samples were obtained from healthy donors under an NIH IRB-approved protocol developed by the Department of Transfusion Medicine, NIH, Bethesda, MD; and HIV-infected patients (N = 25) who were involved in the USAF Natural History Study. All blood samples were collected under protocols that were reviewed and approved by the Institutional Review Boards of the USAF Wilford Hall Medical Center, Lackland AFB, TX and of the National Cancer Institute, Bethesda, MD. Eighteen of the HIV-infected patients were receiving highly-active antiretroviral therapy (HAART), consisting of a combination of two reverse-transcriptase inhibitors and one protease inhibitor, at the time of enrollment. Seven of the HIV-infected patients were HAART-free at the time of enrollment. Plasma viral loads and CD4 counts for all HIV-infected patients included in the study are summarized in Table 1. HAART-treated patients had significantly lower viral load compared to HAART-free patients (P = 0.004), whereas CD4 counts were not significantly different between the two groups (P = 0.808). In vitro experiments were performed using peripheral blood mononuclear cells (PBMC) isolated by density centrifugation using peripheral blood lymphocyte separation medium (Cambrex, Gaithersburg, MD). Cells were cultured in RPMI 1640 (Invitrogen, Gaithersburg, MD) containing 10% fetal bovine serum (Hyclone, Logan, UT) and 1% Pen-Strep-Glut (Invitrogen).

**Table 1:** Patients clinical status

Patient #		CD4 count Cells/µl	VL copies/ml
HAART	1	751	<50
	2	1701	<50
	3	645	<50
	4	948	<50
	5	1059	1770
	6	704	53
	7	616	<50
	8	1056	<50
	9	573	6930
	10	1013	<50
	11	701	<50
	12	648	<50
	13	770	<50
	14	909	<50
	15	342	<50
	16	367	<50
	17	548	54
	18	1112	<50
no HAART	19	846	3370
	20	324	24600
	21	593	1010
	22	618	30800
	23	1296	<50
	24	779	3170
	25	836	30300

### Preparation of noninfectious AT-2 HIV-1

All virus preparations were kindly provided by Dr. Jeffrey D. Lifson, National Cancer Institute, Frederick, MD. HIV-1_MN_ (X4-tropic) and HIV-1_Ada_ (R5-tropic) were inactivated with 1 mM Aldrithiol-2 (AT-2) for 18h at 4°C (AT-2 HIV-1), as described [86]. Microvesicles, isolated from uninfected cell cultures were employed as a negative control [86].

### Stimulation and culture of PBMC

PBMC were cultured with noninfectious AT-2 HIV_MN_, AT-2 HIV_Ada_ or their non-AT-2-treated infectious counterparts at 300 ng/mL p24^CA^ equivalent as previously described [31]. Experiments conducted using AT-2 HIV_MN_, AT-2 HIV_Ada_ or non AT-2-treated infectious HIV-1_MN_ or HIV-1_Ada_ gave comparable results. Only results obtained using AT-2 HIV_MN_ are shown. A mixture of 12 different species of rIFN-α (IFN-α sampler kit, R&D Systems) was used at the final concentration of 1000 U/ml.

### Blocking assays

Blocking of type I IFN receptor was performed by pre-incubating PBMC with 5 µg/ml anti-IFNAR (Invitrogen) for 30 min before addition of AT-2 HIV. Isotype-matched antibodies were used as controls.

### Flow cytometry

After stimulation in culture, cells were washed and incubated for 20 min at room temperature in PBS containing 2% mouse serum (Sigma) with the following antibodies: Peridinn chlorophyll protein (PerCP)-conjugated anti-CD3 (BD Biosciences), Allophycocyanin (APC)-conjugated anti-CD4 (BD Biosciences), PE-Cy7-conjugated anti-CD8 (BD Biosciences), Phycoerythrin (PE)-conjugated anti-CD69 (BD Biosciences), fluorescein isothiocyanate (FITC)-conjugated anti-CD38 (BD Biosciences). Cells were washed twice in ice-cold DPBS and FACS analysis was performed on a FACSCanto flow cytometer using FACSDiva software (BD Biosciences). FlowJo software (Treestar, Ashland, OR) was used to analyze flow cytometry data.

### Inhibition of IDO with 1-methyl tryptophan

The D-isomer of 1-methyl tryptophan (1mT) was used in all IDO-blocking experiments. The D-isomer was chosen because we have previously shown its efficacy in inhibiting HIV-induced degradation of tryptophan and production of kynurenine [32]. 1mT (Sigma) was suspended in deionized water and solubilized by addition of NaOH. HCl was subsequently added to adjust the pH to 7.4. A solution of NaOH and HCl in water prepared in the same conditions as the 1mT was used as negative control.

### CFSE proliferation experiment

CFSE-labeled PBMC from five different donors were cultured in presence or absence of AT-2 HIV as described above, with or without 1-methyl-D-tryptophan (1mT, 200 µM) (Sigma). After 24 hours 1 µg/ml OKT3 anti-CD3 (eBioscience) was added to the cultures. After further 72 hours proliferation was measured by flow cytometry as CFSE dilution in CD3^+^CD4^+^ (CD4 T cells) and CD3^+^CD8^+^ (CD8 T cells) gated PBMC. Calculation of division index (number of cell divisions/total cell number) and proliferation index (number of cell divisions/number of divided cells) was performed using FlowJo software (Treestar).

### Preparation of the two-step experiment

PBMC were isolated from eight different donors. CD4^+^ and CD8^+^ cells were separated from fresh PBMC using anti-CD4 and anti-CD8 magnetic beads (Miltenyi Biotechs, Auburn, CA), according to manufacturer instructions. The remaining PBMC were cultured for 48 hours in presence of control microvesicles, AT-2 HIV or AT-2 HIV plus 1mT at the concentrations described above, while the isolated CD4^+^ and CD8^+^ cells were maintained in RPMI 1640 (Invitrogen,) with 10% fetal bovine serum (Hyclone) and 1% Pen-Strep-Glut (Invitrogen). After 48 hours, the non-adherent cells from CD4^+^ and CD8^+^ cell cultures were collected and purity of CD4 and CD8 T cell population (>98% for both population) and depletion of pDC (undetectable, see Supplemental Figure S1) were determined by flow cytometry. Supernatants from the PBMC cultures were collected and used as conditioned media (CM: control, HIV, HIV+1mT). The autologous CD4 and CD8 T cells were cultured in the three different CM in presence or absence of 1 µg/ml OKT3 anti-CD3 (eBioscience) and 1 µg/ml anti-CD28 (eBioscience). Control experiments were performed by culturing CD4 T or CD8 T cells in fresh media, in presence or absence of AT-2 HIV or AT-2 HIV plus 1-mT, to discriminate between the effect of tryptophan depletion and the cytopathic effect of AT-2 HIV which may still be present in the CM.

### Bioreduction cell proliferation assay

CD4 and CD8 T cells were cultured in the three different CM in presence or absence of 1 µg/ml OKT3 anti-CD3 (eBioscience) and 1 µg/ml anti-CD28 (eBioscience). After 72 hours proliferation was assessed as the increase in the number of viable cells, using the CellTiter 96 AQueous One Solution Proliferation Assay (Promega, Madison, WI), according to the manufacturer's instructions. Titration curves made with serial dilution of cells were not modified by addition of 1-mT, demonstrating that the compound does not influence the assay.

### Annexin V staining

CD4 and CD8 T cells were cultured in the three different CM in presence or absence of 1 µg/ml OKT3 anti-CD3 (eBioscience) and 1 µg/ml anti-CD28 (eBioscience). After 72 hours cells were washed in annexin buffer and incubated for 20 minutes with medium only (negative control) or with fluorescein isothiocyanate (FITC)–conjugated annexin V (Caltag, Burlingame, CA) at room temperature. After 2 washes FACS analysis was performed on a FACSCanto flow cytometer using FACSDiva software (BD Biosciences). FlowJo software (Treestar, Ashland, OR) was used to analyze flow cytometry data.

### Flow cytometry cell cycle analysis

Cell cycle progress of CD4 and CD8 T cells was tested using the FITC BrdU Flow Kit (BD Biosciences), according to the manufacturer's instructions. Briefly, CD4 and CD8 T cells were pulsed for 2 hours with BrdU, then washed fixed and permeabilized before treatment with DNase (1 hour at 37°C) to expose BrdU epitopes. Cells were then washed and incubated with FITC-conjugated anti-BrdU. After the final wash 7-AAD was added to the cells for DNA content staining (all reagents were included in the BD Biosciencesc FITC BrdU Flow Kit). FACS analysis was performed on a FACSCanto flow cytometer using FACSDiva software (BD Biosciences). FlowJo software (Treestar, Ashland, OR) was used to analyze flow cytometry data.

### Quantification of cyclins and CHOP mRNA

Total RNA was extracted from CD4 and CD8 T cells using the guanidium thiocyanate-phenol-chloroform method, modified for TRIzol (Invitrogen, Carlsbad, CA). RNA (1 µg) was reverse transcribed into first strand cDNA by using random hexanucleotide primers, oligo(dT), and Moloney murine leukemia virus reverse transcriptase (Promega, Madison, WI). cDNA quantification was performed by real-time PCR, conducted with an ABI Prism 7900HT (Applied Biosystems, Foster City, CA). All reactions were performed using a SYBR green PCR mix (QIAGEN), according to the following thermal profile: denaturation at 95°C for 15 sec, annealing at 60°C for 15 sec, and extension at 72°C for 15 sec (data collection was performed during the extension step). Primer sequences were designed using the Primer3 software and are presented in Table 2. All results were normalized on GAPDH mRNA expression. Cyclins mRNA results are presented as ratio between anti-CD3/CD28-stimulated cells and unstimulated cells.

**Table 2:** Primers used for real time PCR assays

	Forward	Reverse
**GAPDH**	ccacccatggcaaattcc	tgggatttccattgatgacaag
**Cyclin D1**	ccctcggtgtcctacttcaa	aggaagcggtccaggtagtt
**Cyclin E1**	atcctccaaagttgcaccag	aggggacttaaacgccactt
**CD28**	gtgaaatgctgcagtcagga	gcctgagagtctccgtcatc
**CHOP**	gcgcatgaaggagaaagaac	tcaccattcggtcaatcaga

### Statistical analysis

All in vitro experiments were repeated on PBMC from at least five different donors. Statistical analyses were performed using the SPSS 13.0 software (SPSS Inc., Chicago, IL, USA). Differences between treated and untreated cells were assessed using a two-tailed paired Student's *t*-test. Differences between HIV-1-infected and uninfected donors were assessed using a non-parametric two-tailed Mann-Whitney U test. In all cases *P*<0.05 was considered statistically significant. Univariate distributions of flow cytometric data were performed by probability binning, in 300 bins using FlowJo software.

## Supporting Information

Supplemental Figure S1 Depletion of pDC from CD4+ cells over 48 hours culture. CD4+ cells were isolated from PBMC of HIV-uninfected donors and maintained in culture media for 48 hours before being used in the two-step experiments, as described in Material and Methods. The frequency of pDC (CD123+BDCA2+) was monitored by flow cytometry in the non adherent cells at the time of isolation (0h), after 24 hours of culture (24h) and before use in the two-step experiment (48h). Flow cytometry dot plots show the progressive loss of pDC from the non adherent population over 48 hours.
(2.30 MB TIF)
