# Supplementary figures and images for: HIV-Induced Type I Interferon and Tryptophan Catabolism Drive T Cell Dysfunction Despite Phenotypic Activation
Source: PLoS One. 2008 Aug 13;3(8):e2961. doi: 10.1371/journal.pone.0002961 (PMC2491901; doi:10.1371/journal.pone.0002961)

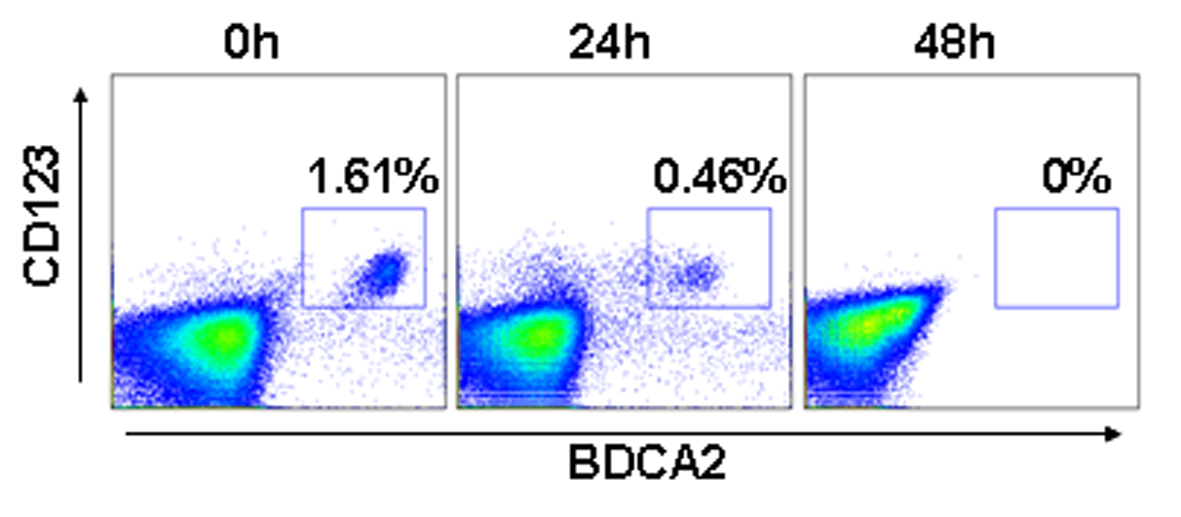

Supplement: Supplemental Figure S1 — Depletion of pDC from CD4+ cells over 48 hours culture. CD4+ cells were isolated from PBMC of HIV-uninfected donors and maintained in culture media for 48 hours before being used in the two-step experiments, as described in Material and Methods. The frequency of pDC (CD123+BDCA2+) was monitored by flow cytometry in the non adherent cells at the time of isolation (0h), after 24 hours of culture (24h) and before use in the two-step experiment (48h). Flow cytometry dot plots show the progressive loss of pDC from the non adherent population over 48 hours. (2.30 MB TIF) [file pone.0002961.s001.tif]
